# Design and validation of a conceptual model regarding impact of open science on healthcare research processes

**DOI:** 10.1186/s12913-024-10764-z

**Published:** 2024-03-07

**Authors:** Maryam Zarghani, Leila Nemati-Anaraki, Shahram Sedghi, Abdolreza Noroozi Chakoli, Anisa Rowhani-Farid

**Affiliations:** 1 Medical Library and Information Sciences, School of Health Management and Medical Information Science, Iran University of Medical Sciences, Tehran, Iran; 2 Department of Medical Library and Information Sciences, School of Health Management and Medical Information Science, Iran University of Medical Sciences, Rashid Yasmin Street, Upper than Mirdamad St., Tehran, Iran; 3 Health Management and Economics Research Center, Iran University of Medical Sciences, Tehran, Iran; 4 Health Management and Economics Research Center, Health Management Research Institute, Iran University of Medical Sciences, Tehran, Iran; 5 Department of Information Science & Knowledge Studies, Shahed University, Tehran, Iran; 6 Department of Pharmaceutical Health Services Research, University of Maryland School of Pharmacy, Baltimore, Maryland USA

**Keywords:** Conceptual model, Open science, Open research, Openness in science, Openness in research, Validation

## Abstract

**Introduction:**

The development and use of digital tools in various stages of research highlight the importance of novel open science methods for an integrated and accessible research system. The objective of this study was to design and validate a conceptual model of open science on healthcare research processes.

**Methods:**

This research was conducted in three phases using a mixed-methods approach. The first phase employed a qualitative method, namely purposive sampling and semi-structured interview guides to collect data from healthcare researchers and managers. Influential factors of open science on research processes were extracted for refining the components and developing the proposed model; the second phase utilized a panel of experts and collective agreement through purposive sampling. The final phase involved purposive sampling and Delphi technique to validate the components of the proposed model according to researchers’ perspectives.

**Findings:**

From the thematic analysis of 20 interview on the study topic, 385 codes, 38 sub-themes, and 14 main themes were extracted for the initial proposed model. These components were reviewed by expert panel members, resulting in 31 sub-themes, 13 main themes, and 4 approved themes. Ultimately, the agreed-upon model was assessed in four layers for validation by the expert panel, and all the components achieved a score of > 75% in two Delphi rounds. The validated model was presented based on the infrastructure and culture layers, as well as supervision, assessment, publication, and sharing.

**Conclusion:**

To effectively implement these methods in the research process, it is essential to create cultural and infrastructural backgrounds and predefined requirements for preventing potential abuses and privacy concerns in the healthcare system. Applying these principles will lead to greater access to outputs, increasing the credibility of research results and the utilization of collective intelligence in solving healthcare system issues.

**Supplementary Information:**

The online version contains supplementary material available at 10.1186/s12913-024-10764-z.

## Introduction

The transformation of information carriers, digital media, and internet tools has created new opportunities for the dissemination and sharing of scientific information, giving rise to the broader concept of open science [1]. Open science aims to take advantage of diverse methods to remove barriers to sharing scientific research [2–4], bringing about fundamental changes in how research is conducted, communicated, published, its results evaluated, researchers collaborated, and scientific works shared [5]. Open science has been recognized as a tool for participatory research management [6]. European Commission has introduced open science as a new approach to scientific processes, which is based on collaborative work and innovative methods of knowledge dissemination through digital technologies [3]. Fundamentally, open science aims to enhance public access to data, analyses, and findings with historical roots. David (1994) suggested that open science likely began during the scientific revolution in 17th century, when printed versions of scientific results were intended for public access [3], implicitly seeking to bridge the gap between science and society through new methods and greater alignment with democratic values and rights as well as promoting access to publicly-funded knowledge and the development of open tools [7].

Given the importance of open science methods and tools in research processes in various fields, many researches have been conducted in this regard. However, most of these studies have focused only on one dimension of various subject areas or one dimension of open science. The highlighted topics include principles and methods of open science in research teams [8], the gap between science and practice in open science [9], open science opportunities in knowledge sharing [10], the relationship between open science policies and research methods [11], clinical data sharing [12], strengthening open science in research process [13], the concept and aspects of open science [3]. In addition, some studies have examined a number of approaches for applying these principles to maximize the value of open science and minimize its adverse effects on the progress of science in practice [8]. For accelerating the dissemination and development of new treatments in neurodegenerative disorders, a new strategy called “Open Science” model has been used experimentally by the Montreal Neurological Institute (MNI) and partners to remove the barriers of many universities and companies [9]. However, in the mentioned study, it has been attempted to identify all aspects of open science that influence the research process as tools and methods promoting and facilitating the research process in the field of health and determine how to use open science methods and tools at each stage of research process in healthcare, including publication, distribution, evaluation and effectiveness of research.

The methods of open science have been clearly effective in the dissemination and access to information in medicine; studies have often focused on methods such as open data, publication of research details, open refereeing, and open research repositories in the organization [12, 14–17]. To maintain the principles of research, ethics and issues such as privacy in the health system should be taken into account in infrastructure and open publishing laws in research organizations and legislative organizations of different countries [18]. Despite recognizing the extensive applications of open science tools in scientific processes, the proponents of open science hold diverse viewpoints on how traditional openness to research outputs should be interpreted [19]. Different definitions, objectives, and commitments have been proposed for utilizing repositories, databases, researcher communication, and open science tools [19]. Substantial variations exist among scientific fields, countries, and stakeholders’ groups regarding open science methods and concepts in relation to policies and program directions [4]. Thus, challenges and opportunities for implementing open science policies in various countries require further investigation and study [3].

Therefore, considering the direct relationship between the method of publishing research outputs, as well as publishing rules, infrastructure and culture governing the subject areas, a specific conceptual framework should be provided to use the open science tools and methods according to the nature of information. The application of open science tools in healthcare system to optimize research outputs for treatment processes, management decisions, and public knowledge enhancement is of high importance [20, 21]. Universities and research centers must address approaches to create value for stakeholders at social, national, and international levels by employing modern technology tools similar to that presented in open science practices to tackle multifaceted challenges. Given this gap, our study aims to identify and validate the influential components of open science on research processes of the healthcare system by using a conceptual model enhancing the understanding of dimensions associated with it for benefiting researchers, policymakers, and healthcare managers. Since open science introduces novel concepts of applicable technologies and innovations in research processes, investigating the implementation of open science methods in research processes of the healthcare system necessitates exploring a conceptual model, which could lead to the formulation of relevant policies, legal conditions for publishing and retrieving various research outputs within the framework of open science for universities and research centers related to the healthcare sector. This conceptual framework is based on an exploratory method, which was conducted by interview, expert panel and Delphi method and presented the effective and important factors in the implementation of open science in health system under the conceptual model.

## Methodology

**Fig. 1:**
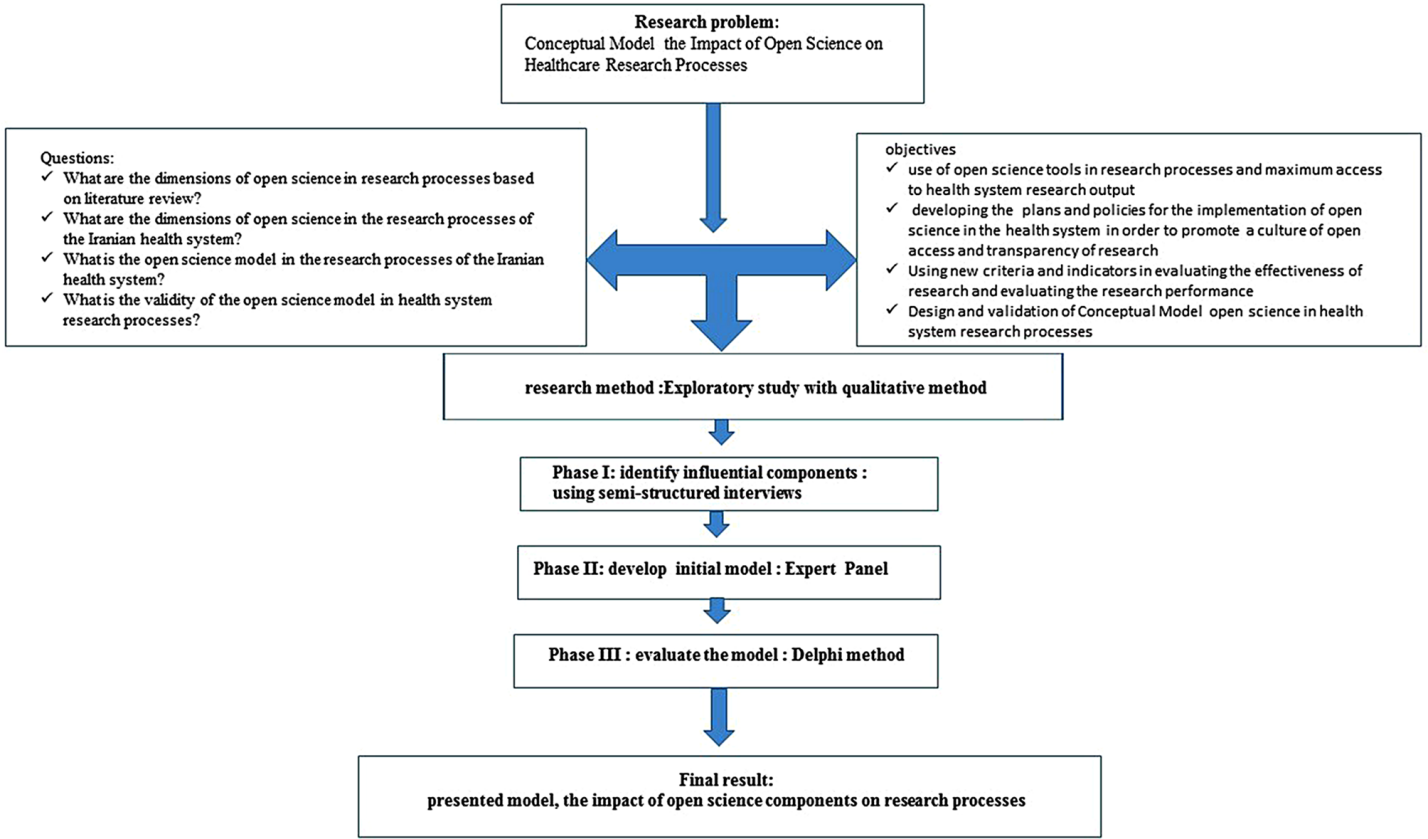
Flow chart of the study design

The current study falls under the category of exploratory research in terms of nature and applied research in terms of research type, which adopts an inductive strategy that show in flow chart of the study desing (Fig. 1). It also utilizes a qualitative data approach by employing a thematic analysis method. To formulate the model components, a three-step process of framework coding was employed. This process involved structuring organized concepts (main themes and subthemes obtained from combining and summarizing codes) and comprehensive concepts (themes encompassing the impact of open science on research process) within the healthcare system with respect to validation purposes (reliability and credibility of themes), for which two methods were utilized. The first method involved communicative validation, meaning referring back to the participants (interviewees) for verification [22]. The second method was expert validation, which utilized expert panels and Delphi technique. Furthermore, for validating the stability of themes, two methods were applied: repeatability and generalizability. The former was achieved through an agreement process between the two coders (i.e., the researcher and a collaborator) regarding coding [23]. This approach aimed to resolve inconsistencies arising from the coding review process. Regarding generalizability, efforts were made to involve various academic and executive stakeholders related to research topic as much as possible. It means that sampling should be done regularly and comprehensively based on the agreement of experts [24].

### Phase I

To identify influential components and develop an initial model, a qualitative method was employed through semi-structured interviews (Appendix 1 & Appendix 2) among academic experts and managers in the field of research and technology within Deputy of Research and Technology of MOHME in different universities. The sampling method was purposeful and snowball, and the individuals needed to meet one of the following criteria: researchers with at least three years of research experience and involvement; academic members or managers who had served in a managerial or executive role in Deputy of Research and Technology within MOHME for at least one semester and were available and willing to cooperate. According to these criteria, interviews continued until data saturation, ultimately resulting in 20 interviews. Data saturation refers to the point where new data on the research topic is no longer obtained during interviews and the data becomes repetitive. The interviews began in early July 2021 and continued until mid-November 2021.

During the first five interviews, initial direction and examination determined the number of questions, timing of interviews, and final interview guidance. Each interview was allotted a time between 40 and 90 min. In the review done by participants to confirm validity, a portion of the text along with initial codes was sent to some of them to compare and validate the coherence of emerging ideas from the data with their own content. In the next step, to control data validity, the method of agreement rate between two coders was employed. Five initial interviews were coded in parallel, and the codes were discussed to reach an agreement. The data analysis method in this phase was the framework analysis. After each interview, the interview was first heard multiple times by the researcher (the one conducting the interview). Then, the text was transcribed using Microsoft Word (version 13) and read multiple times, and initial semantic units were identified. The transcribed files were transferred to MAXQDA software (version 20), and the determination of initial codes and their analysis was performed. The thematic analysis method was used to categorize codes, extract and classify sub-themes and main themes. After analyzing the data, a list of influential components of open science was prepared according to the perspectives of participants, which should be applied in the research processes of healthcare system. This list was used to develop the initial model.

### Phase II

This stage of research was designed according to consensus among experts. The preliminary proposed model was developed based on the components that were extracted from the first stage of the study. Expert panel members were selected using purposeful and available sampling methods. The panel consisted of five research team members, three researchers with research experience in the field of open science and four healthcare system executives related to research and technology. The research was conducted in the workplace of experts using online sessions. In this step, a form designed according to main components and subcomponents was utilized to assess the position of each component in the proposed model considering the experts’ opinions (Appendix 3). The expert panel guidelines were sent electronically and in print to panel members. A one-month time frame was allocated for panel members to complete and review the form. After this period, follow-ups were conducted, both in-person and online, to collect the forms. Once all panel members had submitted their forms, the summarized opinions were entered into the data collection form. To maintain the confidentiality of opinions, they were coded and entered into the form, which was subsequently sent to panel members again with a one-week window for review. In an online session using Google Meet, each component was discussed, and a consensus-based approach was used to confirm the results. Through the review of all components listed in the expert panel guidelines regarding the proposed model, the experts’ opinions regarding the acceptance or rejection of each proposed component were evaluated. Final analysis was performed by assessing each component based on consensus through collective agreement and utilizing the Likert scale. If there was unanimity regarding a component, it was incorporated into the final model. In cases of difference of opinions among the experts, the majority opinion prevailed, leading to revisions and corrections of the component in question.

### Phase III

This stage involved the Delphi method, and the participants were managers and researchers of Ministry of Health who had also participated in the first stage, as well as the activists of the field of open science in MOHME who were invited to evaluate the model. The research sample was selected using purposeful and available sampling methods. In addition, the diversity of participants in this stage contributed to better evaluation and improved the quality of the model. The sample size for this stage ranged from 20 to 30 participants. In the first Delphi round, 24 participants took part, and in the second round, there were 21 participants. To qualify as the study sample, individuals needed to meet at least two of the following criteria: being a faculty member and researcher at one of the medical sciences universities under MOHME, having research or managerial experience in research processes, or being specialists in librarianship and medical information with research experience in open science or having at least three years of active record in research management. The research environment was the workplace of research community members. A structured questionnaire based on the main components and sub-components extracted from interview analysis in the second phase was used for data collection (Appendix 4).

### Implementation process of delphi approach

#### Selection of experts

In studies employing the Delphi method, the sample size varies from 10 to 50 people, which was shown in the study of Campbell and Cantrill [25]. Agumba and Haupt identified 30 experts, out of whom 20 participated in completing the questionnaires [26]. In Rowe and Wrigh analysis of Delphi studies, it has been shown that the number of experts varies from 4 to 21 [27], and Woudenberg stated that he considered between 5 and 20 experts [28]. Based on these references and considering the necessary population size for Delphi studies, the sample size was determined to be between 20 and 30 participants in this study. Experts were selected using purposive sampling. Based on the inclusion criteria explained in the sampling section, the experts at least met two of the conditions for participation. Agumba and Haupt required experts to meet at least three of eight entry criteria [26], while Rogers and Lopez were satisfied with two out of five inclusion criteria [29]. Consequently, 30 experts were first identified and provided with the questionnaire, 24 of whom expressed willingness to participate and took part in validating the model components. Ultimately, the research sample included 24 experts, all of whom were educators and researchers with over three years of research and executive experience.

### Development and validation of questionnaire

Thirteen main themes and 31 sub-themes that were approved by experts as components of the proposed model in the second step were the basis of the closed structured questionnaire design for this step. The first phase analysis and evaluation by the expert panel in the second phase served as the basis for constructing the structured questionnaire for this stage. According to Hsu and Sandford, the use of a closed questionnaire is more appropriate than an open one because a simpler response process and shorter completion time increases the likelihood of greater expert participation [30]. If the members participating in the study are representative of the relevant field of knowledge, it can guarantee the validity of the content [1]. Also, the Delphi approach should not be judged with quantitative methods, but rather transferability, reliability, applicability and confirmability criteria should be considered for the validity and reliability of the results [31]. Since the structured Delphi questionnaire was prepared based on expert panel in the second phase, including representatives from the healthcare knowledge domain and open science practitioners and had also been reviewed by research team as well as some of the participating experts in the third phase, its face validity was confirmed.

### Criterion for achieving consensus

The term “consensus” refers to the agreement on an idea for participants to reach a common ground on a specific topic, rather than finding a correct answer [32]. Research using Delphi method has also shown that there is no specific criterion for achieving consensus. A common criterion in these studies is that at least 60% of respondents should agree on the component under consideration, which occurs with 50–90% probability [32, 33]. Components with agreement levels below this rate are considered not to have reached consensus and move on to the next phase [34]. However, achieving 100% agreement is not feasible due to diverse political, social, economic, and scientific backgrounds of individuals [35]. A decision about consensus is made when a certain percentage of votes fall within a specific range [30]. In previous studies, a consensus range of 51–100% has been reported [36, 37]. In this study, the criterion for achieving consensus for each component was based on research, considering that at least 60% of participants should agree on the importance of the component. Accordingly, responses were scored on a five-point Likert scale, ranging from one to five. The acceptance threshold for each component was a score higher than 75% or > 75% agreement based on the total opinions about it (very much and much). Components that scored between 50% and 75% underwent revisions and were re-entered into the validation cycle for reevaluation. Components that scored < 50% were excluded from the study. The Delphi process was conducted in two rounds to confirm the components. Delphi iterations refer to the process of systematically (and in writing) repeating a series of steps using questionnaires with the aim of reaching consensus on opinions [38]. In terms of the number of iterations, articles have reported 2 to 10 rounds [37]. The decision about the number of rounds is somewhat practical or empirical and depends on available time and the nature of the initial question [37]. In this study, a panel of experts was used for validating the components of the proposed model. As a result, Delphi iterations were implemented in two rounds to validate the components.

### Data analysis

Analysis methods are determined based on Delphi’s objective, the structure of iterations, the type of questions, and the number of participants [30, 38]. Descriptive statistics such as mean, median, and measures of dispersion are commonly used [39]. In this study, descriptive statistics was utilized for analyzing the results of the first and second rounds, including frequency and percentage for ranking the findings. After collecting questionnaires in the first Delphi round, the proposed components were applied, and the results of the first phase along with the revised questionnaire were sent again to study participants. This process continued until consensus was reached on the options. Data analysis in the validation phase was done using descriptive statistics (frequency, percentage), and the responses were scored on a five-point Likert scale. The acceptance criterion for each component was a score > 75%. Components that scored 50–75% underwent revisions and were re-entered into the validation cycle. Components that scored < 50% were excluded from the study.

### Execution of delphi rounds

When necessary information regarding the research topic is available, a structured questionnaire can be used to improve responses [30]. Since the information regarding tool design was obtained in previous steps of this study, a structured questionnaire was used. In the first Delphi round, a total of 30 questionnaires were sent to the identified individuals through e-mail and in-person channels. After two weeks, 24 questionnaires were returned to the research group following repeated follow-ups. At the end of the first round, responses were collected and summarized. The results of this round indicated that there was a consensus of over 75% on 13 main components and 29 sub-components listed in the questionnaire. Two sub-components did not reach consensus in this stage, so the second round of Delphi was initiated. In the Second Delphi Round, the feedback received from the first round along with revisions of components that were not approved was sent to 24 participants of the first round. They were asked to provide their opinions and reasons for agreement or disagreement with the components. After collecting questionnaires in this round and analyzing them, all 31 sub-components and 13 main components achieved a consensus with a score exceeding 75%.

## Results

From the analysis of interviews in the first stage using thematic analysis, a structured collection of 385 codes, 38 sub-themes, 14 main themes, and 3 major themes was extracted. The initial proposed model concerning the impact of open science on health research processes was formed based on the semantic relationship between these components for presentation to the expert panel. Table 1 presents the structured collection of themes, as well as main components, and sub-components extracted from qualitative data related to the interviews.

**Table 1:** Extracted concepts from qualitative interview data

Major themes	Main themes	Sub-themes
Publishing & sharing	Open access to a variety of research outputs	✓ Publishable research items ✓ Access to maximum output ✓ Sharing different data
	Conditions of access to outputs	✓ Access conditions to outputs ✓ User-oriented access level
	Transparency and reproducibility of factors of research credibility	✓ Research reproducibility ✓ Research transparency
	Channels of publishing and sharing outputs	✓ Informal channels for publishing research output ✓ Formal channels for publishing research output ✓ Publishing modes of the results for the public
	Citizens’ participation in research stages	✓ Participation in all stages of research ✓ Knowledge cycle and research credibility
Infrastructural and cultural	Infrastructure-tools for registration & sharing	✓ Tools for recording and sharing research cases ✓ Data publishing infrastructure ✓ Library for open-research management and publishing
	Management and protection infrastructure	✓ Research stages’ management platform and system ✓ Data publishing protocol ✓ Protective infrastructure
	Culturalization and education	✓ Transparency culture ✓ Educating the principles of open-science ✓ Educational and culturalization requirements
	Formation of extensive scientific communications	✓ Extensive research collaborations ✓ Communication paths ✓ New communication tools
	Publishing costs	✓ Citizens’ participation in research budgets ✓ Adjustment of publication costs
Monitoring and evaluation	Legislation and guidelines	✓ Facilitating the intellectual property of research ✓ Rules and mechanisms of open research
	Ethical principles in the research process	✓ Organizational monitoring of open-research process ✓ Institutionalization of research ethics ✓ Ethical considerations in publishing data
	Supportive policies	✓ Research budget transparency ✓ Organizational support ✓ Executive and incentive policies
	Open-research evaluation process	✓ Open peer review of articles ✓ Research efficiency ✓ Research evaluation indicator ✓ Supervisory Working Group

In the second phase, to review and refine the titles of extracted components and the semantic relationships established between them in the proposed model according to experts’ opinions, the model was evaluated and reviewed by experts using the data collection form (Appendix 3). The summary of experts’ opinions that aimed at revising, refining the titles, and establishing semantic relationships between the proposed model’s initial components indicated a collective agreement on most of the components. Furthermore, summarizing experts’ opinions and applying them to the proposed model led to the refinement and enhancement of components. The modified titles of the components were as follows: “Enhancing Factors of Trust in Research Outputs,” “Publishing Peer-Reviewed Results and Other Outputs in Scientific Networks,” “Publishing Research Outputs in the Scientific Language,” “Disseminating Research Outputs to the Public,” “Enhancing Participation in All Research Stages,” “Increasing Public Involvement in Data Collection,” “Strengthening the Knowledge Cycle and Trust in Research,” “Leveraging Innovative Communication Tools,” “Public Participation in Research Funding,” “Mechanisms and Guidelines for Open Research,” “Facilitating Intellectual Property Conditions for Research,” and “Promoting Ethical Principles in the Research Process.”

Semantic congruence according to expert opinions and reevaluation of codes and components led to the integration of eight components as follows: “Publishable Research Topics,” “Publishing Research Outputs in the Scientific Language,” “Infrastructure and Tools for Sharing Outputs,” “Protective Infrastructure and Data Sharing,” “Training in Open Science Principles,” “Ethical Considerations in Publishing Outputs,” “Supportive and Encouraging Policies,” and “Evaluation Indicators.” Based on the revisions suggested, new concepts emerged during the re-review of codes and component meanings: “Transparency of Research’s Scientific and Technical Process,” “Transparency of Research’s Managerial and Financial Process,” “Impact of Open Science on Regulatory Processes,” “Impact of Open Science on Evaluation Processes,” and “Open Peer Review.”

**Fig. 2:**
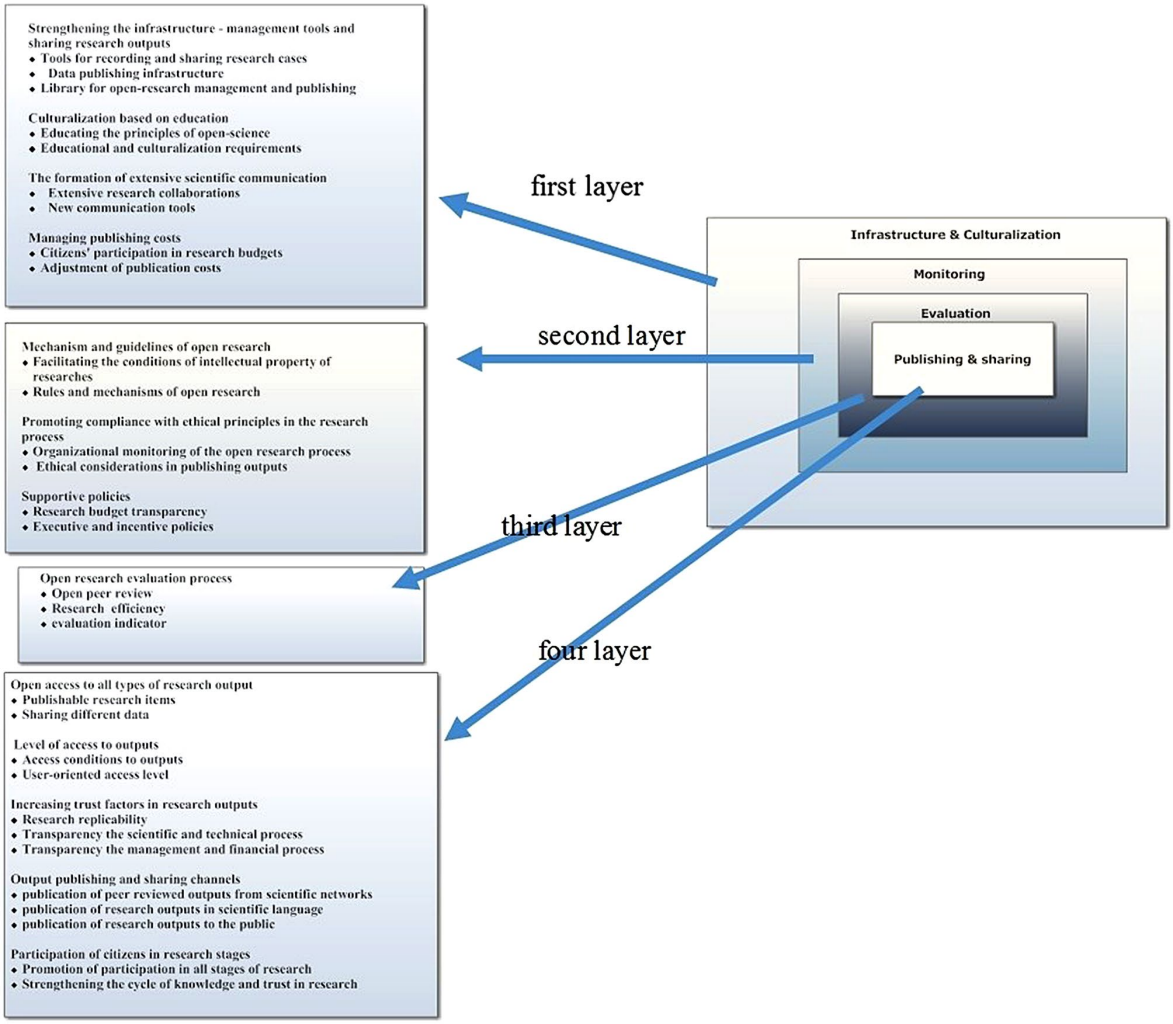
Conceptual model of the impact of open science on research processes in healthcare system of Iran

According to the overall opinion, open science is considered an effective factor in reducing research barriers. A uniform research structure cannot be proposed for all organizations. The sub-component “Unified Form of Open Research Structure” was removed. The initial coding was also reviewed again. Applying the suggestions received from experts led to reconsideration of the initial proposed model. Ultimately, the proposed model was selected for final evaluation using Delphi method, which consisted of 31 sub-components, 13 main components, and 4 super-components as shown in (Fig. 2).

The results of final stage of the study, which aimed at validating the proposed model regarding the impact of open science on research processes of the healthcare system, were obtained using the classical Delphi method and quantitative descriptive statistics. Participants of this stage consisted of 13 males (54.2%) and 11 females (45.8%). All the participants (100%) had more than five years of research experience. According to the opinions of participants in the first round of Delphi, all sub-components, main components, and super-components (a total of 31 sub-components, 13 main components, and 4 super-components) reached a consensus except for two sub-components, namely “Transparency of Managerial and Financial Process of Research” and “Publishing Research Outputs to the Public”. The acceptance or rejection of each component depended on the total opinions received (very much and much) and required a score of > 75%.

The second round of Delphi was conducted to reevaluate the two components that could not achieve a score > 75%. Accordingly, a questionnaire was designed for the participants to assess the impact of these two components on research process in the second round of Delphi. The questionnaire was sent to 24 participants who took part in the first round. After analyzing the data from this stage, these two components also reached a consensus with a score > 75%.

All components of open science that were effective on research processes of healthcare system reached a consensus in both rounds of the Delphi process. Table 2 categorizes the importance levels of the main components according to opinions of Delphi study participants into three levels. Four components, namely “Enhancing Trust Factors in Research Outputs,” “Mechanisms and Guidelines for Open Research,” “Promoting Ethical Principles in the Research Process,” and “Open Research Evaluation Process” achieved 100% consensus among participants. Additionally, three components, including “Formation of Extensive Scientific Communication,” “Managing Publication Costs,” and “Supportive Policies,” were ranked second with over 90% agreement. Components that ranked third also obtained consensus with over 80% agreement. This indicates the importance of all components in the proposed model and the need for proper implementation of each.

**Table 2:** Ranking of main themes of open science affecting research processes in the healthcare system

Rank	Main themes	Importance					Sum total/ percentage	Collective agreement/ percentage
		Very- low (F/P)	Low (F/P)	Medium (F/P)	Much (F/P)	Very- much (F/P)		
1	Increasing trust factors in research outputs	0 (0)	0 (0)	0 (0)	5 (8/20)	19 (2/79)	24 (100)	24 (100)
	Mechanism and guidelines of open research	0 (0)	0 (0)	0 (0)	8 (3/33)	16 (7/66)	24 (100)	24 (100)
	Promoting compliance with ethical principles in the research process	0 (0)	0 (0)	0 (0)	5 (8/20)	19 (2/79)	24 (100)	24 (100)
	Open research evaluation process	0 (0)	0 (0)	0 (0)	7 (2/29)	17 (8/70)	24 (100)	24 (100)
2	The formation of extensive scientific communication	0 (0)	0 (0)	1 (2/4)	6 (25)	17 (8/70)	24 (100)	23 (95/8)
	Managing publishing costs	0 (0)	0 (0)	1 (2/4)	6 (25)	17 (8/70)	24 (100)	23 (95/8)
	Supportive policies	0 (0)	0 (0)	1 (2/4)	3 (5/12)	20 (3/83)	24 (100)	23 (95/8)
	Strengthening the infrastructure - management tools and sharing research outputs	0 (0)	0 (0)	1 (2/4)	6 (25)	17 (8/70)	24 (100)	23 (95/8)
	Output distribution and sharing channels	0 (0)	0 (0)	2 (3/8)	6 (25)	16 (7/66)	24 (100)	22 (91/7)
	Open access to all types of research output	0 (0)	0 (0)	2 (3/8)	5 (8/20)	17 (8/70)	24 (100)	22 (91/6)
	Culturalization based on education	0 (0)	1 (2/4)	1 (2/4)	5 (8/20)	17 (8/70)	24 (100)	22 (91/6)
3	Level of access to outputs	1 (2/4)	0 (0)	2 (3/8)	8 (3/33)	13 (2/54)	24 (100)	21 (87/5)
	Participation of citizens in research stages	0 (0)	1 (2/4)	2 (3/8)	11 (8/45)	10 (7/41)	24 (100)	21 (87/5)

^1^Frequency/Percent

In the presented model, the impact of open science components on research processes is structured in four main layers, forming the foundation for open research policy. This model, which is derived from analysis of interviews and expert opinions relevant to research topic, created a structure leading to open research policy. In the first layer, namely the broadest layer, the necessary hardware and software equipment for implementing open science research methods should be provided, as along with issues such as specialized human resources, technical infrastructure, software, systems, and tools needed for conducting research in an open manner, as well as pathways for sharing, which should be taken into account. The educational principles required for fostering open science culture are considered in this layer, too. The second layer is essential for determining the necessary principles and strategies for implementing open science research. In this layer, the laws, ethical principles in open research and policies are determined; it is a fundamental step towards creating an open research policy and plays a role in all stages of research. The third layer is based on open peer review, research efficiency, and evaluation indicators related to pre- and post-publication evaluation of research results, as well the impact of research from various aspects, which should be measured based on quantitative and qualitative indicators. The fourth layer is related to the process of publishing and sharing of research outputs addressing publishable aspects of research, access principles and conditions, transparency and reproducibility processes of open research. Additionally, pathways for accessing research outputs and participation of citizens are defined in this layer. For the establishment of this layer, previous layers must be systematically and effectively defined and supported. The proper formation of these four layers will lead to an open research policy for health system research, resulting in better issue identification, transparent process execution and responsiveness of research, as well as effective utilization of outputs by relevant stakeholders.

## Discussion

Open science can have a significant impact on various research processes. By providing an integrated digital research structure, it can facilitate broader access to outputs and increase participation in various research stages, fostering interactions among researchers and stakeholders within academic, industrial, and policy-making structures. In some cases, open science can be likened to a double-edged sword. On one hand, it could be constructive and transformative, while on the other, it might create challenges such as privacy concerns and the lack of protection for stakeholders’ rights. Nevertheless, the application of open science methodologies in health system research is both constructive and advantageous, with its benefits potentially outweighing the drawbacks. For this purpose, it is necessary to utilize the unique opportunities of open science to enhance knowledge and science derived from research through a specific perspective and plan. This would lead to knowledge democratization and proper utilization in various societal strata, alongside increased community awareness and appropriate utilization of research outputs. Consequently, open science methodologies play a pivotal role in quality management of science. As a result, this support will lead to a win-win situation [40].

Whereas the use of these technologies has led to challenges in some cases, the potential and actual benefits have been so impressive that newer measures should be taken to apply these technologies correctly. One of the goals of organizational science is contributing to evidence-based development in problem solving. Since studies such as clinical trials and cohorts in the field of medical sciences are looking for a scientific and practical basis in the direction of evidence-based medicine, the use of open science methods in these research processes to discover and test evidence-based actions can be beneficial for doctors [20]. One of the most prominent advantages of open science in healthcare system is providing conditions for maximum public access to scientific outputs in an understandable language free from complexity. Utilizing diverse scientific discourse methods through various media outlets should be considered in this regard. Nonetheless, for proper utilization of research outputs to create conducive conditions, the need for a cycle of credible and transparent knowledge circulation arises. And a well-established knowledge cycle based on sharing outputs across different research stages enhances trust in research structures, fosters greater participation, and ultimately amplifies the impact of research across different societal domains. To fulfill these requirements, various dimensions of open science provide this crucial opportunity to researchers and stakeholders, yielding significant cost-effectiveness for institutions and universities [13]. An open science research policy comprises scientific dissemination channels, participation, university relationships, research quality and coherence, transparency, repeatability, requirements for transparent scientific processes, and a system for alignment and evaluation [6]. This system is achievable based on values of openness, fair sharing, resource accessibility, education of research outputs, and acceptance of open culture [41]. Therefore, an open science platform should have several properties, including categorizing multiple versions of data and codes, supporting multiple data access schemes, especially for sensitive data, flexible metadata management and standards in evolution, connecting organizational and external data, supporting object identifiers such as DOI, facilitating internal and external scientific collaboration and participation [36]. These characteristics enable the digital support of all research steps within the framework of open science.

Models of open access and open data dissemination are rapidly becoming open scientific methods that influence the entire research ecosystem, including production, communication, and reuse of research results. Utilizing technological innovations for the dissemination of scientific content is vital for sustainability of scientific journals and publishers [42]. Nevertheless, in the current context, these practices are not widely adopted because insufficient knowledge on utilizing these practices, potential misuse of research, imposing high publication costs on researchers, and so forth have led to negative reactions towards the application of these methods. Also, the lengthy process of open peer reviews and the dissemination of evaluation feedback have not been favorable for researchers. Appropriate policies with clear mechanisms are needed to create desirability and confidence among stakeholders for conducting research within the framework of open science; for example, encouraging factors and preventive measures against potential misuse. Transparency and openness in research require cultural transformation. Enhancing transparency and openness should not only be embraced by scientists and researchers, but also by budget-providing institutions and even those beyond the research and innovation sector [43]. Moreover, the budgetary mechanism for publishing research outputs plays a crucial role in this stage. Most academics support the principle of making knowledge freely available to everyone, but the use of open access publications among academics is still limited due to relevant policies [44]. Additionally, legal and ethical issues in research have prompted the development of new tools and methods for addressing these matters. European Commission has deemed the implementation of open science processes as a task for universities to free themselves from these conditions [45].

### Limitations

Due to their nature, qualitative studies have limitations. It has been attempted to reduce these limitations with the measures taken for validity and reliability of the study as follows. Some participants were not willing to cooperate in the interview when the purpose of the research was explained to them and they were assured that their information would remain confidential. The time and place of the interview was determined according to their wishes. In addition, the timetable to conduct this study was arranged according to the communication restrictions imposed by COVID-19, which caused the time to collect and carry out various stages of the study to be longer than usual. In order to solve this limitation, reminders were sent via e-mail, as well as face-to-face and telephone follow-ups to receive comments. The diversity and geographical dispersion of participants was another issue that caused a lot of time to follow up and receive information. An attempt was made to use auxiliary forces in different geographical areas of Iran to follow up and receive information.

## Conclusion

The conceptual model presented based on the findings of this study has shown that to apply open science methods in different stages of research in the health system, it is essential to cultivate a culture of open research and ethical issues through formal and informal education or repeated communication within universities and research centers, which reaches various stakeholders. The technical infrastructure should also be established, which has already been provided to a considerable extent in research libraries through monitoring software of research centers and universities. Access conditions should be reconsidered based on the type of research and the target audience. Another important finding based on this model was that the laws and policies for implementing open research in the healthcare system should be formulated through university research councils and ethics committees, so that the support of higher-level organizations and lawmakers, as well as necessary laws are enacted and enforced. Additionally, principles and assessment processes must consider various aspects such as effectiveness, problem-solving, participation and collaboration in different projects, as well as transparency enhancements. Based on the conditions and processes outlined in different layers of this model, maximal dissemination and sharing of various research outputs will result in the greatest degree of research application in healthcare system and various strata of society.

In general, the findings of this research have shown that open science methods can be highly effective in improving the research process and benefiting from its outputs, which requires providing sufficient background, knowledge and skills to apply each of them in different stages of research. In line with the findings of this study, it is suggested that the organizations in charge of health system should review research guidelines and communication processes between research stakeholders. And in connection with the influential factors in cultural processes, infrastructure and supervision help implement the open research process by forming specialized working groups consisting of people active in the field of research, observing ethics in research, evaluation and validation of studies, as well as knowledge translation groups. The principles of transparency and scientific openness by research organizations and universities should be considered as a codified and strategic plan because it will cause positive consequences, including increasing the amount of scientific credibility, widespread participation of different people in research, and benefiting more from the scientific knowledge produced. Also, to create an organizational culture based on the results obtained in the policy department, it is suggested that the principles of scientific openness should be considered as an aspect of research activities of organizations and universities. Considering the importance of the type of data and research outputs in health system and privacy protection, openness and open access to research results can be defined according to the type of studies. And the tools and services that provide the conditions of scientific openness should be defined as one of the strategies of organizations and universities because open science accelerates the conditions for creating a culture of scientific openness in organizations. According to the necessity of open research topic in future studies, open science should focus on the following topics. Compilation of open research evaluation principles based on new indicators in the health system, presenting a user model to apply each of the open science methods in health system research, the effect of teaching necessary skills to apply open science methods by researchers and research supporting organizations, compilation of ethical principles and adjustment of intellectual property in health system researches, compilation of the conditions of access to information and data of health system with an emphasis on privacy and biosecurity issues. With the identification of these factors, the research stakeholders will proceed to widely use open science methods in a safer intellectual environment.

### Electronic supplementary material

Below is the link to the electronic supplementary material.

**Supplementary Material 1:** Inductive interview guideline

**Supplementary Material 2:** Informed consent form

**Supplementary Material 3:** A tool for collecting experts’ opinions in the second step to modify the initial coding and the proposed model

**Supplementary Material 4:** A tool for collecting experts’ opinions in the third step for evaluation of the proposed model

## Data Availability

The datasets formed and analysed during the current study are available from the corresponding author on reasonable request.
