# Supplementary material for: Design and validation of a conceptual model regarding impact of open science on healthcare research processes
Source: BMC Health Serv Res. 2024 Mar 7;24:309. doi: 10.1186/s12913-024-10764-z (PMC10921571; doi:10.1186/s12913-024-10764-z)
Supplement: Supplementary file 1 — Supplementary Material 1: Inductive interview guideline [file 12913_2024_10764_MOESM1_ESM.docx]

**Additional File 4**

**Subject:** A tool for collecting experts' opinions in the third step for evaluation of the proposed model

**Objective**: Evaluation of the validity of the proposed model of open science in the research processes of the health system (Delphi rounds)

The following questionnaire has been compiled as the third step of the study to evaluate and determine the importance of the influencing components of open science on research processes in the health system. It is worth mentioning that this study titled "**Developing an conceptual model for open science in health system research processes**" has been registered in Iran University of Medical Sciences and Health Services and has received an ethics certificate with code **IR.IUMS.REC.1399.462**. Therefore, at this stage of the study, by identifying the influencing components of open science on research processes in the health system, the importance of each component is tried to be investigated. For this purpose, you are invited, if possible, to give your opinions regarding each of the components according to the Likert scale (very much (5), much (4), medium (3), low (2), very low ( 1)) specify. The main components are marked with gray color and the * sign, and the related sub-components are listed below each one. If you have any suggestions other than those presented, please include them at the end of the questionnaire. In addition, in order to understand the importance and position of each of the components, the initial proposed model, which was formed based on the opinions of experts, is presented at the end. It should be mentioned that in order to preserve the identity information of the participants, comments will be used anonymously.

| Demographic information  Gender: male and female  Degree: Master's degree  Scientific rank: ... research work experience ... executive work experience: ... | | | | | | |
| --- | --- | --- | --- | --- | --- | --- |
| **The impact of open science in the research process** | Definition | Importance | | | | |
|  |  | Very much | much | moderate | low | Very low |
| ***Open access to all types of research output** |  |  |  |  |  |  |
| Publishable research items |  |  |  |  |  |  |
| Sharing different data |  |  |  |  |  |  |
| ***Level of access to outputs** |  |  |  |  |  |  |
| Access conditions to outputs |  |  |  |  |  |  |
| User-oriented access level |  |  |  |  |  |  |
| ***Increasing trust factors in research outputs** |  |  |  |  |  |  |
| Research replicability |  |  |  |  |  |  |
| Transparency the scientific and technical process |  |  |  |  |  |  |
| Transparency the management and financial process |  |  |  |  |  |  |
| ***Output publishing and sharing channels** |  |  |  |  |  |  |
| publication of peer reviewed outputs from scientific networks |  |  |  |  |  |  |
| publication of research outputs in scientific language |  |  |  |  |  |  |
| publication of research outputs to the public |  |  |  |  |  |  |
| ***Participation of citizens in research stages** |  |  |  |  |  |  |
| Promotion of participation in all stages of research |  |  |  |  |  |  |
| Strengthening the cycle of knowledge and trust in research |  |  |  |  |  |  |
| **The impact of open science on infrastructure process - research culture** | Definition | Importance | | | | |
|  |  | Very much | much | moderate | low | Very low |
| ***Strengthening the infrastructure - management tools and sharing research outputs** |  |  |  |  |  |  |
| Tools for recording and sharing research cases |  |  |  |  |  |  |
| Data publishing infrastructure |  |  |  |  |  |  |
| Library for open-research management and publishing |  |  |  |  |  |  |
| ***Culturalization based on education** |  |  |  |  |  |  |
| Educating the principles of open-science |  |  |  |  |  |  |
| Educational and culturalization requirements |  |  |  |  |  |  |
| ***The formation of extensive scientific communication** |  |  |  |  |  |  |
| Extensive research collaborations |  |  |  |  |  |  |
| New communication tools |  |  |  |  |  |  |
| ***Managing publishing costs** |  |  |  |  |  |  |
| Citizens' participation in research budgets |  |  |  |  |  |  |
| Adjustment of publication costs |  |  |  |  |  |  |
| **The impact of open science on the monitoring process of research** | Definition | Importance | | | | |
|  |  | Very much | much | moderate | low | Very low |
| ***Mechanism and guidelines of open research** |  |  |  |  |  |  |
| Facilitating the conditions of intellectual property of researches |  |  |  |  |  |  |
| Rules and mechanisms of open research |  |  |  |  |  |  |
| ***Promoting compliance with ethical principles in the research process** |  |  |  |  |  |  |
| Organizational monitoring of the open research process  Ethical considerations in publishing outputs |  |  |  |  |  |  |
| ***Supportive policies** |  |  |  |  |  |  |
| Research budget transparency |  |  |  |  |  |  |
| Executive and incentive policies |  |  |  |  |  |  |
| **The impact of open science on the research evaluation process** | Definition | Importance | | | | |
|  |  | Very much | much | moderate | low | Very low |
| ***Open research evaluation process** |  |  |  |  |  |  |
| Open peer review |  |  |  |  |  |  |
| Research efficiency |  |  |  |  |  |  |
| evaluation indicator |  |  |  |  |  |  |
