# Supplementary material for: Design and validation of a conceptual model regarding impact of open science on healthcare research processes
Source: BMC Health Serv Res. 2024 Mar 7;24:309. doi: 10.1186/s12913-024-10764-z (PMC10921571; doi:10.1186/s12913-024-10764-z)
Supplement: Supplementary file 2 — Supplementary Material 2: Informed consent form [file 12913_2024_10764_MOESM2_ESM.docx]

**Additional file 1**

**Inductive Interview Guideline**

**Title**: **Developing an conceptual model for open science in health system research processes**

**Open science:** It is an approach to the scientific process based on collaborative work, with new tools and ways to collaborate and disseminate knowledge through online digital technologies. And its main purpose is obvious sharing and reuse of scientific knowledge created by researchers as a result of publicly funded research projects so that it leads to public access and reuse of scientific data, transparency in scientific communication, use of web-based tools to facilitate academic collaboration, ensuring openness principles to the entire research cycle from beginning to the end steps, strengthening knowledge sharing and collaboration among scientists.

**Questions**

1. Tell me about your research and work field in the health system (education level, job position, research tasks, and field)

2. What is your opinion about open methods in the research process?

3. What is your definition of open science or openness in research? What are its applications in health related research?

4. What are the stages of publishing research outputs in the health system? How has open science or openness affected these processes?

5. What do you think about the transparency of research processes? How important is transparency in health research?

6. How do you define research replicability? What is its significance?

7. What effect does community participation have on health research?

8. How open scientific communications are formed in research? How do you interact with other people in the research community (reviewers, other researchers, and research collegues)?

9. What infrastructure is needed for open-research? What tools and services are needed to share, communicate, and collaborate on open-research in the health system?

10. What should the budget mechanism look like? How can be the participation of community members in the research budget process?

11. How can we promote the culture of open-research?

12. What are the requirements and policies for the spread of open-research and monitoring processes in the health system? (laws, principles, technology, monitoring processes, and costs of implementation and publication)

13. How should be the monitoring open-research process? That is, on what basis and principles can the correct process of open-research be designed? (organizational governance, copyright laws (to facilitate and protect with open-access), research committees, and ethics in organizations)

14. How do you think the effectiveness and use of open-research should be evaluated?

15. In general, what do you think should be included if you consider a framework for open-research?

16. Is there anything else not mentioned by you in this conversation and you wish to express it?
