# Supplementary material for: Design and validation of a conceptual model regarding impact of open science on healthcare research processes
Source: BMC Health Serv Res. 2024 Mar 7;24:309. doi: 10.1186/s12913-024-10764-z (PMC10921571; doi:10.1186/s12913-024-10764-z)
Supplement: Supplementary file 3 — Supplementary Material 3: A tool for collecting experts’ opinions in the second step to modify the initial coding and the proposed model [file 12913_2024_10764_MOESM3_ESM.docx]

**Additional 2**

**Informed Consent Form**

Informed consent letter for the research on “ **”** as part of the doctoral dissertation as **"Developing an conceptual model for open science in health system research processes".**

I, (participant's name in full) ___________________ agree to participate in the mentioned research project between 2019 and 2021 that was approved by the Medical Ethics Committee of Iran University of Medical Sciences [date: Jul 2020, ID: IR.IUMS.REC.1399.462].

I understand that the text and recorded verbal or electronic communications with the researcher will be studied and may quote excerpts in a doctoral dissertation and in future articles, journal articles, and books that the researcher may write. I understand that my responses will be anonymous and that no identifying (personal) information will be disclosed in any written or verbal context.

I understand that my participation is completely voluntary and that I may withdraw from the study without explanation at any stage if I do not wish to continue.

Name: ................................................

Address: ..........................

Signature: ................................................

Date : .........................

Signature of the researcher: .........................

Date: ..........................

**Researcher: Maryam Zarghani, 09023532972, mary.zarghani@gmail.com**

**Department of Library and Medical Information, Faculty of Management and Medical Information,**

**Iran University of Medical Sciences.**

Please keep a copy of this information for yourself and give one copy to the researcher.
