# Supplementary material for: Design and validation of a conceptual model regarding impact of open science on healthcare research processes
Source: BMC Health Serv Res. 2024 Mar 7;24:309. doi: 10.1186/s12913-024-10764-z (PMC10921571; doi:10.1186/s12913-024-10764-z)
Supplement: Supplementary file 4 — Supplementary Material 4: A tool for collecting experts’ opinions in the third step for evaluation of the proposed model [file 12913_2024_10764_MOESM4_ESM.docx]

**Additional 3**

**Project Details**

**Subject**: A tool for collecting experts' opinions in the second step to modify the initial coding and the proposed model

**Objective**: Designing an open science model for use in processes and publishing the maximum output from research in the health system

Emphasizing the importance and fair dissemination of research outputs, for access to documented, clear and approved knowledge to promote and improve the welfare of societies, the process of conducting research should be transparent and maximum access to their output, especially those that are funded with public funds, should be provided in the form of open access. The use of new communication and information tools has led the research process to digital and often open access. Open research (open science) is proposed as an umbrella that includes all research processes in a digital format, and its purpose is to provide conditions for maximum access to research output, transparency and better accountability to the society. So that the conditions of cooperation and participation of most people in the society are provided, data and other research processes are freely available, and it allows the distribution, use and reproduction of data and research methods to improve the quality of science through clarification and reproducibility. In this regard, the following questionnaire is designed and submitted for the third step of my doctorate thesis on medical library and information science with the title "**Developing an conceptual model for open science in health system research processes**". This thesis is registered in Iran University of Medical Sciences and Health Services and has received the ethics certificate with code **IR.IUMS.REC.1399.462.**

In the frist step of this research, interviews were conducted with 20 health researchers with research experience. The approved texts were coded and analyzed using MAXQDA software version 20, finally to design the initial model of 3 main themes that were formed based on the goals of this step and the open research policy in the health system that was obtained based on the analysis of texts and interviews. From the 385 codes obtained from the analysis of the text of the interviews, 38 sub-categories and 14 main categories were presented for 3 themes of the process of publishing and sharing, infrastructure and culture, monitoring and evaluation of open research in the health system and open research policy.

In this step, we try to extract the main and subcategory titles based on your comments and examine their position in the proposed model. If possible, you are requested to specify your opinion about the title of each of the roles, their place in the proposed model, overlapping with other roles, correct grouping of roles under each main category, etc. The main categories are marked with gray color and * in the table, and related subcategories are listed below each one. In order to preserve your identity information, your comments will be used anonymously.

| Publication and sharing of research according to open science | | The degree of agreement with the proposed role  (The results of the analysis of the interviews ) | | | | | **Offers**  (Regarding the simplicity and understanding of the role, the definition of the role, the position of the role in the model, overlapping with other roles, the correct grouping of roles or the correlation of roles under each main category, etc.) |
| --- | --- | --- | --- | --- | --- | --- | --- |
| The role of open science in the research process | Role definition | completely agree | agree | refused | disagree | completely disagree |  |
| *open access to a variety of research outputs | open access to the article, final report and other items such as research data, tools and software, details of the research process, especially in laboratory studies and issues that must be done under certain conditions and standards under the title of documenting the process, details and conditions for It is intended to conduct research. |  |  |  |  |  |  |
| Publishable research items | The output is in the form of an article or final report, research protocols in review works or clinical trials, documentation, research details and even an operational plan that is developed to conduct a research. |  |  |  |  |  |  |
| Access to maximum output | Access to the most output apart from the final findings or article includes data, software, details and method etc. |  |  |  |  |  |  |
| Sharing different data | Sharing and access to all types of data, including research, treatment, public and government data and other data that are necessary for research, decision-making and policy-making in the field of health. |  |  |  |  |  |  |
| *Dissemination process and access level to outputs | In accordance with a specific protocol or charter, in which the level of access and the amount of sharing are determined according to the type of research and its outputs. |  |  |  |  |  |  |
| access conditions to outputs | According to the conditions of the type of research, the audience, the time dimension and other influential factors, how to access the outputs is determined. |  |  |  |  |  |  |
| User-oriented access level | The conditions of access to the outputs are appropriate for specific audiences, this process is carried out by special working groups and at each stage of the research, they can monitor how the outputs are shared and specify the access conditions. |  |  |  |  |  |  |
| *Trancparency and replicability of credibility factors | Relying on research outputs for application, decision-making requires their clarification and reproducibility, which is achieved by accessing and publishing data, details, methods, other research outputs and reports to specific audiences, and leads to better research evaluation and conditions. |  |  |  |  |  |  |
| Research replicability | The conditions of re-doing the study in the same conditions and confirming the results, which has led to the validity of the results and the evaluation, testing and more detailed examination of the outputs and provides the background for secondary studies and researchers, in addition to learning and reducing previous errors and mistakes. |  |  |  |  |  |  |
| Research trancparency | Clarification by publishing detailed reports, recording and publishing the details of the research to the researcher, supporting organizations and other members of society shows the honesty and trust of the research and provides the basis for reproducibility. |  |  |  |  |  |  |
| *Publishing and sharing channels of outputs | The ways of publishing outputs in the process of conducting research from official ways such as journals, organizational archives and informal ways like scientific social networks (academia, research gate, etc.) and public media (television, radio, etc.) for the access of experts and non-experts (community people) is considered. |  |  |  |  |  |  |
| Informal channels for publishing research output | The way of publication of outputs and communication channels is through scientific networks such as Academia, Research Gate, Mendeley, Twitter, etc., through which the results of the research can be published in scientific and non-scientific language for the majority of society. |  |  |  |  |  |  |
| Formal channels for publishing research output | The official channels for the publication of research outputs are often the findings through scientific journals, organizational and academic archives, library portals that publish the research results in scientific language for peers, professionals and the academic community. |  |  |  |  |  |  |
| Publishing paths of the results for the public | Public access to research outputs through mass media such as television, radio, public magazines, public and organizational websites, even scientific and public networks such as LinkedIn, Mendeley, Twitter, WhatsApp, etc., considering that most people in the society The media has access. |  |  |  |  |  |  |
| *Citizens’ participation in research stages | It is related to the two concepts of citizen science and crowding resources in health system research. Based on the participation of citizens in different stages of research, from the initial stages of idea formation, carrying out, publishing, republishing and using findings, providing funding, evaluating and criticizing the outputs, they can have different roles. |  |  |  |  |  |  |
| Participation in all stages of research | In health research, citizens have the ability to participate in all stages of research, from identifying problems, forming ideas and research topics, conducting, publishing, republishing and using findings, providing funding, evaluating and criticizing outputs, which creates trust and companionship in research, and a sense of self-esteem. And it becomes useful in society. |  |  |  |  |  |  |
| Participation in data collection | Most of the health system researches are based on population data from the level of different societies. The participation of citizens in the form of data collection in the form of voluntary contributions and even by using data collection platforms based on population work has provided the conditions for the participation of many people from the society in the research. |  |  |  |  |  |  |
| Knowledge cycle and research credibility | The cycle of knowledge based on the publication and sharing of research outputs among the people of the society from two classes of experts and the general public leads to better trust and communication with the results of studies and conditions for better influence of the outputs. |  |  |  |  |  |  |
| Infrastructure - open science culture for the research process | | The degree of agreement with the proposed role  (The results of the analysis of the interviews ) | | | | | **Offers**  (Regarding the simplicity and understanding of the role, the definition of the role, the position of the role in the model, overlapping with other roles, the correct grouping of roles or the correlation of roles under each main category, etc.) |
| The role of open science in the research process | Role definition | completely agree | agree | refused | disagree | completely disagree |  |
| *Registration and subscription infrastructure and tools | The necessary tools for a research to be accessible and protected from the beginning are archives, organizational repositories, library portals, journals, research systems such as clinical trials in the world, and technical infrastructure platforms (frameworks) such as equipment, knowledge and specialized personnel. |  |  |  |  |  |  |
| Tools for recording and sharing research cases | Tools such as organizational archives or repositories in universities and research centers, library archives and document centers, library portals, research systems, etc. |  |  |  |  |  |  |
| Data publishing infrastructure | Publishing and sharing data must be under a suitable, coherent and trackable platform (framework) that can provide appropriate and audience-oriented access conditions and protect the rights of the beneficiaries, which requires technical equipment, knowledge and expert human resources. |  |  |  |  |  |  |
| Library for open-research management and publishing | Based on the technical infrastructure and specialized forces embedded in the libraries, it enables the organization and dissemination of information, and it is a suitable place for open research to manage, record, publish and share the outputs as much as possible and protect them. |  |  |  |  |  |  |
| *Management and protection infrastructure of research stages | Platforms and systems that provide technical and software infrastructure for managing, protecting and tracking research outputs, such as the European Commission's open science framework, or clinical trial systems that can be registered and accessible in these systems from the beginning of the proposal design. |  |  |  |  |  |  |
| Research stages management platform and system | Creating a coherent and unified framework for all types of research in the health system that researchers and organizations can implement, record and share their research according to it for their specific audiences at every stage of the research. It means that the researchers face a ready structure and in that structure they can carry out and publish their research work. |  |  |  |  |  |  |
| Data publishing protocol | According to the importance and value of data in the health system, a suitable protocol or format for data dissemination should be determined, and this format provides accessibility for the audience and data protection conditions according to the type of data and research work. |  |  |  |  |  |  |
| Protective infrastructure | An infrastructure that determines in what structure open research should be conducted and how it can be followed up and monitored after publication. |  |  |  |  |  |  |
| *Culturalization and education | Cultivation of open research requires education and awareness based on publishing brochures, training courses and learning ethical issues, which is achieved by implementing these things in organizations, universities and research centers and reflecting them in the society and providing appropriate information among the people of the society. |  |  |  |  |  |  |
| Transparency culture | The culture of transparency is created in accordance with the correct training of open research and the creation of conditions for access to research details for audiences at different levels. |  |  |  |  |  |  |
| Educating the principles of open-science | Getting to know and understand the rules and conditions of open research for researchers and other stakeholders by providing content and holding appropriate workshops in this connection forms the preparation and encouragement towards the implementation of open research in the society. |  |  |  |  |  |  |
| Educational and culturalization requirements | Educational and cultural requirements should be provided by upstream organizations such as the Ministry of Health, universities and research centers that are in charge of health system research in accordance with the specific types and conditions of medical studies and the qualifications of individuals to conduct open research. |  |  |  |  |  |  |
| *Formation of extensive scientific communications | Scientific communication is the main element of any research, which is formed from the preparation stage to the final report and products with various people from the research team and outside the research team such as peers, evaluators, and finally, almost the entire society is involved for its use and effectiveness. In open research, this relationship should be determined effectively. |  |  |  |  |  |  |
| Extensive research collaborations | In the structure of open research, due to the fact that all stages of research can be openly and transparently available to its specific audiences, it creates a wide scientific space and increases cooperation at the local, national and international levels. |  |  |  |  |  |  |
| Communication paths | Communication channels in the traditional form are related to journals, conferences, reports and books, organizational repositories, etc., which publish the results of studies. In the new way, the website of journals, virtual conferences, e-mails and online scientific networks, the website of organizations, scientific and library portals, repositories and online archives. |  |  |  |  |  |  |
| New communication tools | Modern communication tools are often related to scientific networks, websites, online archives, magazine websites, e-mail, etc., and one of the most important channels of informal scientific communication is online scientific networks such as Research Gate, Academia, Mendeley. |  |  |  |  |  |  |
| *Publishing costs | Often publishing through open access journals has a fee, so this fee must be paid by the researcher or organization in charge of the research. |  |  |  |  |  |  |
| Citizens' participation in research budgets | Non-governmental organizations, philanthropists and various people of the society can participate in funding the research to carry out and publish the outputs. |  |  |  |  |  |  |
| Adjustment of publication costs | Through methods such as supportive and encouraging policies, financial support of the organization, reducing the costs of publishing journals, special privileges for researchers and research topics that have worked, taking into account special privileges by journals for those who have collaborated with the journal, and using public donations for Publication of research outputs is possible. |  |  |  |  |  |  |
| Monitoring and evaluation of open science on the research process | | The degree of agreement with the proposed role  (The results of the analysis of the interviews ) | | | | | **Offers**  (Regarding the simplicity and understanding of the role, the definition of the role, the position of the role in the model, overlapping with other roles, the correct grouping of roles or the correlation of roles under each main category, etc.) |
| The role of open science in the research process | Role definition | completely agree | agree | refused | disagree | completely disagree |  |
| *Legislation and guidelines | For the implementation of open research in the health system, it is necessary that the laws, mechanisms and executive instructions and the necessary requirements to carry out this process are first specified by the relevant managers and the necessary policies are made. |  |  |  |  |  |  |
| Adjusting the intellectual property of research | In the new and open structure for sharing the maximum outputs, there is a need to adjust and facilitate the intellectual property laws by complying with the appropriate requirements, so that the meaning of this ownership and rights can be clarified for the beneficiaries in different classes. |  |  |  |  |  |  |
| Rules and mechanisms of open research | The requirements and rules for open research should be considered at the macro level and the micro level. At the macro level, managers and policy makers should determine these laws that are enforceable. At the micro level, researchers, community members and supporting organizations should implement the laws. |  |  |  |  |  |  |
| *Ethical principles in the research process | Ethics in research in the field of health is discussed in terms of the type of information, the privacy of individuals and the use of research outputs that directly and indirectly affect the health of society, that is, access to the most outputs and their use, respect for rights, identity information of individuals and the effectiveness of research results. The society should be considered in this structure. |  |  |  |  |  |  |
| Organizational monitoring of open-research process | Educational and research organizations in ethics committees in research should determine the principles and rules of open research in a legal and systematic framework and get the approval of the parent organization of the Ministry of Health, and finally monitor and follow up their implementation by these organizations. |  |  |  |  |  |  |
| Institutionalization of research ethics | Creating research ethics and accountability to all stakeholders in front of what is done and the claims related to outputs so that this thinking is formed in the essence of researchers and people involved in research. |  |  |  |  |  |  |
| Ethical considerations in publishing data | Necessary considerations according to the type of data based on ethical principles and rules of open research should be designed and revised and create bilateral conditions that identity information is protected and maximum access to data types is provided for different people. |  |  |  |  |  |  |
| *Supportive policies | Open research requires comprehensive support from researchers and stakeholders, including in the aspects of access to resources, software, the process of recording and publishing outputs, costs, consulting and expert guidance in line with open research. |  |  |  |  |  |  |
| Research budget transparency | Clarifying the amount and how to spend the budget should be clearly defined for the public and relevant people, the ability of people, priorities and needs should be taken into account in budget allocation, which requires the support of budget allocating organizations and researchers. |  |  |  |  |  |  |
| Organizational support | Related to matters such as financing costs, adjusting publishing and sharing costs, providing educational and awareness fields, conditions for sharing and recording outputs, using information technology and librarians to register, publish and protect, manage and categorize them. , creating technical infrastructure, systems, etc. |  |  |  |  |  |  |
| Executive and incentive policies | Related to issues such as awards, allocation of more funds, providing organizational support, considering special points in the evaluation of researchers and researches, considering all outputs in the evaluation such as the value that is considered for articles, data and other outputs are also considered. be done, including the principles for follow-up and the rights of the beneficiaries and.. |  |  |  |  |  |  |
| *Open-research evaluation process | From the aspect of efficiency and its impact in society, improving the state of society, projects based on needs and using the outputs for other researches, clarifying the different stages of research, sharing the outputs through different channels, etc. are discussed. |  |  |  |  |  |  |
| Research efficiency | The effectiveness of researches is related to things such as the definition of projects based on health system issues and problems, the conditions and extent of using outputs in different sectors, solving health system problems, etc. |  |  |  |  |  |  |
| Research evaluation factors | A total of quantitative and qualitative indicators such as the number of shareable datasets, the amount of presence in scientific networks and knowledge exchange, the amount of international cooperation in terms of scientific communication in these networks, the transparency of different research processes, honesty and correctness in doing work, Elmatrix indicators, considering Expert opinions and evaluations are desired. |  |  |  |  |  |  |
| Supervisory Working Group | A supervisory working group consisting of human forces specialized in ethics committees or evaluation committees that can review open research in terms of the process, compliance with laws and ethical principles, effectiveness and evaluation method. |  |  |  |  |  |  |
